# Editorial: Recent Advances of Epigenetics in Crop Biotechnology

**DOI:** 10.3389/fpls.2016.00413

**Published:** 2016-03-31

**Authors:** Raúl Álvarez-Venegas, Clelia De-la-Peña

**Affiliations:** ^1^Unidad Irapuato, Centro de Investigación y de Estudios Avanzados del Instituto Politécnico NacionalGuanajuato, México; ^2^Centro de Investigación Científica de Yucatán, Unidad de BiotecnologíaMérida, México

**Keywords:** epigenetics, crops, agricultural, biotechnology

Ever since the first Agricultural Revolution, humans have domesticated hundreds of plant species and it is considered that the evolution of crop plants took place as human behavioral ecology changed from food gathering to farming. As a result of wild species domestication, the selection of populations with desirable alleles, the breeding of high yielding genotypes, the ease of farming and quality, and many technological advances have allowed crop production to increase and become better adapted to environmental changes (Bennett et al., 2013). During the last decades, modern breeding methods, as well as novel research, development, and new technologies have improved considerably agriculture production worldwide. This has been achieved thanks to the enhancement of agronomic traits such as, increased abiotic/biotic stress tolerance, reduced toxicity, bigger seed size, increased yield, superior nutritional quality, delayed ripening, better post-harvest quality, etc. (Meyer and Purugganan, 2013). However, due to an increasing human population, nowadays arable soil is becoming less available and the climate change problem is a worldwide emergency.

Plants are indispensable in our life because they supply us with oxygen, food, and medicines. Therefore, it is important to study and examine the mechanisms that plants have evolved to adapt to diverse environments, and in particular how crop species deal with different types of biotic and abiotic stress. In addition, it is essential to understand how the epigenetic component regulates plant gene expression and the plant phenotype, and we must focus on how the epigenome works as a powerful source of diversity for important agronomical traits and on how its exploitation, in crop improvement programs, would be of benefit to our modern society.

It is now known that epigenetic modifications control gene expression by modulating the access of regulatory complexes to the genome. Furthermore, current research on epigenetic mechanisms indicate that DNA methylation, histone posttranslational modifications and small non-coding RNAs are involved in almost every aspect of plant life including agronomically important traits such as flowering time, fruit development, responses to environmental factors, and plant immunity. Even though the basic epigenetic mechanisms in crop biotechnology are starting to be uncovered, soon they will be extensively employed for crop improvement and to increase crop productivity in challenge environments. This research Topic includes an excellent combination of Mini Reviews, Reviews, Original Research Articles, and Methods focused on the role of epigenetics in crop biotechnology, and provides up-to-date information on epigenetics in crop plants during *in vitro* culture, abiotic and biotic stresses, and gene silencing.

One of the biotechnological tools for crop improvement has been the use of plant *in vitro* culture and their effects on epigenetic mechanisms. For instance, in their Original Research article Barraza et al. show that down-regulation of the *PvTRX1h* gene (which codes for a histone lysine methyltransferase, HKMTase) is accompanied by an altered concentration of distinct plant hormones in common bean embryogenic calli. Specifically, “*PvTRX1h* regulates the expression of genes involved in auxin biosynthesis, and embryogenic calli, in which *PvTRX1h* is down-regulated, are able to differentiate into and overproduce somatic embryos.” Also, down-regulation of *PvTRX1h* increase transcript abundance of *PvASHH2h*, a gene coding for a second HKMTase, and point out that epigenetic changes such as histone methylation have an active role in the regulation of plant hormone biosynthesis in common bean calli. Specific topics on the role of chromatin modifications in plant somatic embryogenesis (SE) are discussed by De-la-Peña et al. and provide interesting new insights into the field. In their Review they highlight recent discoveries on the mechanisms of epigenetic regulation in SE that could help to increase plant productivity and improve agronomical breeding practices. On the other hand, Kitimu et al. analyzed epigenetic changes during propagation by meristem culture and by field cuttings in cassava (*Manihot esculenta*) cultivars. They identify candidate epimarks that distinguish between field cutting and meristem culture samples. This will certainly help in the identification of specific methylation signatures associated to *in vitro* propagation and in the optimization of *in vitro* meristem propagation protocols and in the diagnosis of the origin of clonal stocks.Chávez-Hernández et al. report on the miRNA abundance and their target gene expression in response to light exposure and hormone depletion during maize SE. They find that most of the miRNA examined increase upon hormone depletion, regardless of photoperiod absence/presence, whereas expression of miRNA target genes is effectively regulated by the photoperiod exposure. Furthermore, “stress-related miRNA targets show greater differences between cultivars than development-related targets, with a miRNA/target inverse relationship more frequently observed in darkness than light.” Such results will help to understand and manipulate the plant regeneration process in crops like maize. Also, the effect of light was studied in the Original Research article of Liu et al. who show that in rice the enhancer of zeste [E(z)] genes *SDG711* and *SDG718*, are involved in the regulation of key flowering genes and imply that “Polycomb Repressive Complex2 (PRC2)-mediated epigenetic repression of gene expression is involved in the accurate photoperiod control of rice flowering.” Although miRNAs have been analyzed mainly by Northern blots, Rosas-Cárdenas et al. show that tissue-printing hybridization is very useful for detection and localization of miRNAs in fruits of crop plants.

Crop agriculture has two major problems, which are deficiency in nutrient and in water supply, and different authors address these challenges. Firstly, in their Review, Paul et al. highlight the role of miRNAs in macro- or micro-nutrient deficiencies in plants, and how miRNA-mediated regulation of nutrient transporters and other metabolic enzymes could be used in future biotechnological research. Bocchini et al. investigate the effect of iron deficiency, in barley plants, on plant growth and using gene silencing and the changes in the DNA methylation status caused by Fe deprivation, amongst other traits. They find a clear effect of Fe starvation on the level of DNA methylation/demethylation of the barley genome. This kind of research will certainly help to elucidate “how the plants modulate gene expression to cope with nutrients fluctuations,” considering that such modifications could be transmitted to progeny. On the other hand, Su et al. find, in peanut (*Arachis hypogaea*), an RPD3/HDA1-like superfamily histone deacetylase (HDAC), termed AhHDA1, which is seemingly involved in the epigenetic regulation of stress resistance genes in response to osmotic stress and ABA treatment. Accordingly, studies on the molecular mechanisms of drought resistance are necessary and could be used to generate new crop varieties for agriculture in water-limiting conditions. Rodríguez López and Wilkinson review current knowledge on epigenetic states (in particular DNA methylation) and responses of crop plants to specific characteristics of the growing environment (epigenetic fingerprinting) that could be used for the improvement of crop production and quality. More specific topics on genotype × environment interactions that may be beneficial for long-term improvement of crop performance are addressed by King. In his Review, King addresses the biophysical and thermodynamic properties of DNA, histones and nucleosomes, and explores the consequences of thermal and ionic variation on the biophysical behavior of epigenetic marks and how these contribute to maintenance of chromatin integrity and gene regulation in the plant nucleus. Loza-Muller et al. describe how the *Brassica oleracea* fibrillarin methyltransferase is capable to methylate nucleolar histone H2A while bound to the rDNA and carry out its methylation in the rDNA promoter. But not only abiotic conditions affect epigenetics and therefore plant behavior, also biotic challenges are an important topic of study. In their Review Article, Ding and Wang draw attention to the molecular mechanisms of histone modifications and chromatin remodeling that contributes to plant immunity against pathogens. Also, Hohn put emphasis in his Mini Review on the RNA-based silencing suppression mechanisms in plant pararetroviruses. In Meyer et al. the authors analyze the effects of the ectopic overexpression of the *Arabidopsis Enhancer of RNAi* (*ERI*) gene and the link between plant growth and siRNAs. In their Mini Review Rajeevkumar et al. examine the field of epigenetic silencing in transgenic plant systems.

The Research Topic presented here is significant because it is expected to increase and strengthen the information needed to develop, in the near future, novel approaches to manipulate and selectively activate, and/or inhibit gene expression, proteins and metabolic pathways to counter plant pathogens, to treat important diseases and to increase crop productivity. New approaches of the type presented here and the advancement of new technologies will certainly increase our knowledge of currently known epigenetic factors and chromatin modifications and will facilitate the understanding of their roles in, for example, host-pathogen interactions and crop productivity.

## Author contributions

RA and CD provided the idea of the work. RA and CD critically reviewed the manuscript. RA wrote the paper. All authors read and approved the final manuscript.

## Funding

This work was supported by “CONACYT” grant CB-2015 #257129 to RA, and grant #178149 to CD-l-P.

### Conflict of interest statement

The authors declare that the research was conducted in the absence of any commercial or financial relationships that could be construed as a potential conflict of interest.

## References

[B1] Bennett A. E. Daniell T. J. White P. J. (2013). Benefits of breeding crops for yield response to soil organisms, in Molecular Microbial Ecology of the Rhizosphere, Vol. 1 and 2, ed de Bruijn F. J. (Hoboken, NJ: John Wiley & Sons, Inc.), 17–27.

[B2] Meyer R. S. Purugganan M. D. (2013). Evolution of crop species: genetics of domestication and diversification. Nat. Rev. Genet. 14, 840–852. 10.1038/nrg3605 24240513

